# Supplementary material for: Effect of attention on human direction-discrimination thresholds at iso-eccentric locations in the visual field: A registered report protocol
Source: PLoS One. 2023 Nov 30;18(11):e0289411. doi: 10.1371/journal.pone.0289411 (PMC10688726; doi:10.1371/journal.pone.0289411)
Supplement: Attachment — Submitted filename: PLOS_Preregistered protocol.pdf [file pone.0289411.s001.pdf]

This is an interesting study and I recommend that it is conducted, taking into account my following comments.
Signed, Marisa Carrasco

Lines 17-20 (Abstract) / Lines 40-42. (Introduction) *"In humans, the resulting spatial profile of perceptual performance (the "performance field") typically shows better performance in the lower visual hemifield, compared to the upper hemifield, and better performance along the vertical meridian compared to the horizontal meridian."* (This information is repeated in the Introduction section; lines 40-42).
There are two issues: (1) the term 'performance field' usually refers to better performance along the horizontal than the vertical meridian (opposite to what is stated), and better performance along the lower than upper vertical meridian (not visual hemifield as stated).

Lines 43-44.*These inhomogeneities have been speculated to have a retinal or cortical origin, i.e. to reflect sensory inhomogeneities [1-4,7-10].*
There are better references for retinal factors–Kupers et al. 2020, 2022– and for cortical factors –Silva et al., 2018; Benson et al., 2021; Himmelberg et al. 2022, 2023.

Lines 45-48. *Multiple studies report that attention enhances performance, sensitivity, and thresholds homogenously across all locations in the visual field. It is important to note that this is not always the case [3,5,6,11,12], raising the possibility that attention can compensate for some of the inhomogeneities.*
There are no citations for attention's homogeneous effect, and it should be noted that this is the case for *discriminability*; here are some references for spatial attention: Carrasco et al., 2001; Cameron et al., 2022; Purokayastha et al., 2021; Talgar & Carrasco 2002; Roberts et al., 2016, 2018.
Minor point - instead of enhancing thresholds it should say decreasing thresholds
Regarding the references noting that it this is not always the case, please note that:
Ref.3 is about appearance, not discriminability
Ref.5 is about speed of information accrual, not discriminability
Ref.6 reports advantages for motion, when distractors are present, in the two quadrants (not along the vertical meridian) of the lower visual field
Ref.11 reports asymmetries between the upper and lower hemifields related to the observation that the upper and lower visual fields seem to specialize for processing distant and near objects, respectively, but with regard to attention there does not seem a consistent bias to the upper or the lower hemifield (see pages 526-527)
Ref.12 the authors *infer* attention (from a feature- vs a conjunction-detection task); there is no comparison between focal and distributed attention conditions. These stimuli also differ in other ways, e.g., the features consist of tilt lines and the conjunctions of horizontal and vertical lines. In the crowding and 'attention tracking' experiments the stimuli are along the hemifields. Subjects were instructed to fixate in the center, but there was not eye tracking.

Lines 49-50. There should be an explicit motivation for using a dynamic stimulus to investigate visual field asymmetries, which mostly have been investigated using static stimuli.
Note that evidence for performance fields with motion discrimination is weak. For example, a recent study reports no such asymmetries (Ezzo et al., JoV 2023, p.13). If the primary goal is to further investigate whether attention has an effect on performance fields, it would be good to consider using a stimulus that will guarantee them. Else, the possibility that there may be no visual field asymmetries, but that it would still be important to evaluate whether attention effects are homogeneous or not at different polar angles should be discussed.

Lines 94-95. *Each trial will begin with a participant directing his/her gaze at the central fixation cross and pressing the starting key.* It would be good to start the trial contingent on gaze; this seems to be implied but is not explicit.

Line 100: 100% valid post-cue; it is better to refer to it as a ‘response cue’ (also in Lines 131 and 142)

Speed will likely be an important factor in this study, but was not specified in the methods; different underlying mechanisms have been reported for fast and slow speeds (e.g., Giaschi et al., 2007)

Would ‘feedback’ be provided?

Lines 114-115. *The motion directions of a distractor will be the picked randomly from the two staircase values for that location.* Instead of ‘a distractor’, it should say ‘each of the three distractors’, right?

Lines 133-135. Participants will then need to respond, first by reporting the location of the target stimulus and then by reporting the directional tilt (clockwise or counter-clockwise) (fig 2A,B).
It is unclear why participants need to first report the target stimulus location before reporting the directional tilt. It could be confusing to press the location button (right) first and then have to choose between both buttons to indicate direction (on half the trials, FIG 2B). Partial overlap of the buttons in 2B and 2C could be confusing; better to be both different or both the same). The two-stage response and their overlap would prevent analysis of RTs, which can be analyzed to rule out speed-accuracy trade-offs. Why not give both responses by making only one keypress, out of 4 pairs of keys (see for example, Liu Fuller & Carrasco, 2006).
Fig 2B-C could be read as indicating that HM and VM will be tested separately, but according to information on Line 156 all 4 locations will be interleaved in a block.

Lines 153-154. Note that 640 successive trials in one block in the main experiment is very likely to cause fatigue in observers; 5 or 6 blocks each 120 trials, as the number of trials per block in the practice session, sounds more reasonable.

Line 163 mentions Fig 4A depicts H1 and H0, but it does not. Figure 4B depicts the hypotheses. It would make more sense to first show hypotheses about performance fields and then about attention effects; i.e. 4B should be 4A and vice versa.

More detail on the psychometric function fits would be good. For example: How many parameters? Will the upper and lower asymptote be free parameters, or fixed at 0 and 1 like in the figure? This point matters because the parameter of interest is derived from a distance between two predetermined points, it will depend in part on the asymptotes (as well as the slope).

Lines 165-166. These two hypotheses will be tested separately for each of the four cardinal directions.
With regard to direction, I recommend exploring (a) a 2-way interaction of cardinal direction X location-RDP. For example, it is more likely that performance field asymmetries emerge when the reference axis is horizontal than vertical because overall performance would be expected to be higher at horizontal than vertical meridians and there is a slight radial/tangential asymmetry (Ezzo et al., 2023); (b) a three-way interaction among location X direction X attention condition.
