# Supplementary material for: Effect of attention on human direction-discrimination thresholds at iso-eccentric locations in the visual field: A registered report protocol
Source: PLoS One. 2023 Nov 30;18(11):e0289411. doi: 10.1371/journal.pone.0289411 (PMC10688726; doi:10.1371/journal.pone.0289411)
Supplement: Attachment — Submitted filename: Response to Reviewers.docx [file pone.0289411.s003.docx]

Rebuttal letter for the manuscript PONE-D-23-01491 entitled “Effect of attention on human direction-discrimination thresholds at iso-eccentric locations in the visual field: a registered report protocol”

Responses to reviewer #1

1) *In the general field of open science, you should provide detailed information to facilitate replicability of the study. It seems counter-intuitive for science, but recent papers suggest that replicability of methods is lacking. See Asendorpf et al., 2016 for details.*

- We thank reviewer #1 for pointing us to the publication Asendorpf et al. 2016. Our registered report protocol follows the recommendations of Asendorpf et al. 2016. To improve on this issue, we now provide details on the sample size decisions, stimulus details (speed of motion) and behavioral paradigm (absence of feedback) in the revised manuscript. Together the provided information should allow anybody to replicate our study.

2) *In the participant section, please insert a power analysis in order to demonstrate that the sample size is adequate. If you use a mixed linear model for the analysis (see below), please also use this approach in this part (I suggest using the simr package in the R statistical environment).*

- We revised the manuscript according to this recommendation.

3) *Include in the description of participants the "final sample size", but for transparency, all discarded participants should be reported with the reason for their removal. Take into account that removing some participants is normal (participants who did not complete all sessions, inaccurate measurements, etc.), but more importantly, all data removed needs to be reported for transparency, along with the criteria used.*

- The reviewer raised an important point. We always planned to report all the data, including the data from the removed subjects. A corresponding statement is now added in the participant section in the revised manuscript. We will include all the information, including the reasons for the removal of subjects, in a table in the appendix.

4) *I recommend inserting a schematic representation of the overall procedure (session and block) after figure 3*

- Figure of the revised session plan has been added as figure 3.2.

5) *In the analysis section I suggest using the Linear Mixed Model (LMM) instead of GLM. I suggest using ID as a random factor.*

- Thank you for the recommendation. We will indeed use a mixed model. In addition to the individual ID, we include a further random intercept effect of ‘test day’, nested in the individual ID effect, which accounts for potential day-to-day variations in the individuals’ discrimination thresholds. Furthermore, we will include all theoretically identifiable random slopes, as their inclusion has been shown to be important to avoid an overconfident model and keep type I error rates below nominal 0.05 (Barr et al., 2013; Schielzeth & Forstmeier, 2009). This is now described in the data analysis section of the revised manuscript.

Responses to Reviewer #2

1) *Lines 17-20 (Abstract) / Lines 40-42. (Introduction) “In humans, the resulting spatial profile of perceptual performance (the “performance field”) typically shows better performance in the lower visual hemifield, compared to the upper hemifield, and better performance along the vertical meridian compared to the horizontal meridian.” (This information is repeated in the Introduction section; lines 40-42).
   There are two issues: (1) the term ‘performance field’ usually refers to better performance along the horizontal than the vertical meridian (opposite to what is stated), and better performance along the lower than upper vertical meridian (not visual hemifield as stated).*

- Thank you for catching this mistake. We have corrected it.

2) *Lines 43-44. These inhomogeneities have been speculated to have a retinal or cortical origin, i.e. to reflect sensory inhomogeneities [1-4,7-10].
   There are better references for retinal factors–Kupers et al. 2020, 2022– and for cortical factors –Silva et al., 2018; Benson et al., 2021; Himmelberg et al. 2022, 2023.*

- Thank you for the references provided. We have added them in the revised manuscript.

3) *Lines 45-48. Multiple studies report that attention enhances performance, sensitivity, and thresholds homogenously across all locations in the visual field. It is important to note that this is not always the case [3,5,6,11,12], raising the possibility that attention can compensate for some of the inhomogeneities.
   There are no citations for attention’s homogeneous effect, and it should be noted that this is the case for discriminability; here are some references for spatial attention: Carrasco et al., 2001; Cameron et al., 2022;* *Purokayastha et al., 2021; Talgar & Carrasco 2002; Roberts et al., 2016, 2018.
   Minor point - instead of enhancing thresholds it should say decreasing thresholds
   Regarding the references noting that it this is not always the case, please note that: Ref.3 is about appearance, not discriminability
   Ref.5 is about speed of information accrual, not discriminability
   Ref.6 reports advantages for motion, when distractors are present, in the two quadrants (not along the vertical meridian) of the lower visual field
   Ref.11 reports asymmetries between the upper and lower hemifields related to the observation that the upper and lower visual fields seem to specialize for processing distant and near objects, respectively, but with regard to attention there does not seem a consistent bias to the upper or the lower hemifield (see pages 526-527) Ref.12 the authors infer attention (from a feature- vs a conjunction-detection task); there is no comparison between focal and distributed attention conditions. These stimuli also differ in other ways, e.g., the features consist of tilt lines and the conjunctions of horizontal and vertical lines. In the crowding and ‘attention tracking’ experiments the stimuli are along the hemifields. Subjects were instructed to fixate in the center, but there was not eye tracking.*

- Thank you for pointing out that the references for attention’s homogenous effect was missing. The two out of four citations were in the reference list. The list has been revised and citations are added.
- The minor point about enhancing thresholds has been resolved in the manuscript.
- Additional information about the references has been added.

4) *Lines 49-50. There should be an explicit motivation for using a dynamic stimulus to investigate visual field asymmetries, which mostly have been investigated using static stimuli.
   Note that evidence for performance fields with motion discrimination is weak. For example, a recent study reports no such asymmetries (Ezzo et al., JoV 2023, p.13). If the primary goal is to further investigate whether attention has an effect on performance fields, it would be good to consider using a stimulus that will guarantee them. Else, the possibility that there may be no visual field asymmetries, but that it would still be important to evaluate whether attention effects are homogeneous or not at different polar angles should be discussed.*

- The aforementioned studies have observed the presence of attentional inhomogeneities that, to some extent, compensate for sensory inhomogeneities across the visual field. However, the majority of these studies employed static stimuli, with only one exception: the study by Rezec and Dobkins (2004), which used dynamic stimuli placed in the four quadrants. Consequently, no previous research has investigated the compensation of sensory inhomogeneities with attentional inhomogeneities for dynamic stimuli placed along the cardinal meridians. Given the findings of Ezzo et al. (2023), we can anticipate the presence of weaker or smaller attentional inhomogeneities, as dynamic stimuli tend to generate smaller sensory inhomogeneities.
- The study by Ezzo et al., 2023 does point to a very crucial aspect in motion direction discrimination that is the sensitivity to the motion direction is asymmetric and there are three such asymmetries – greater sensitivity to the motion direction is present for cardinal to oblique, moderate sensitivity for polar cardinal to oblique, and small sensitivity for radial to tangential directions. Keeping in mind the findings of this study is crucial to interpret the results of our study.

5) *Lines 94-95. Each trial will begin with a participant directing his/her gaze at the central fixation cross and pressing the starting key. It would be good to start the trial contingent on gaze; this seems to be implied but is not explicit.*

- The sentence has been revised.

6) *Line 100: 100% valid post-cue; it is better to refer to it as a ‘response cue’ (also in Lines 131 and 142) Speed will likely be an important factor in this study, but was not specified in the methods; different underlying mechanisms have been reported for fast and slow speeds (e.g., Giaschi et al., 2007). Would ‘feedback’ be provided?*

- We are now mentioning that we use a dot speed of 3 degs/second.
- We now point out that no feedback is provided.

7) *Lines 114-115. The motion directions of a distractor will be the picked randomly from the two staircase values for that location. Instead of ‘a distractor’, it should say ‘each of the three distractors’, right?*

- Yes. The sentence has been revised.

8) *Lines 133-135. Participants will then need to respond, first by reporting the location of the target stimulus and then by reporting the directional tilt (clockwise or counter-clockwise) (fig 2A,B).
   It is unclear why participants need to first report the target stimulus location before reporting the directional tilt. It could be confusing to press the location button (right) first and then have to choose between both buttons to indicate direction (on half the trials, fig 2B). Partial overlap of the buttons in 2B and 2C could be confusing; better to be both different or both the same). The two-stage response and their overlap would prevent analysis of RTs, which can be analyzed to rule out speed-accuracy trade-offs. Why not give both responses by making only one keypress, out of 4 pairs of keys (see for example, Liu Fuller & Carrasco, 2006). Fig 2B-C could be read as indicating that HM and VM will be tested separately, but according to information on Line 156 all 4 locations will be interleaved in a block.*

- Thank you for this great suggestion. We have replaced the gamepad with a keyboard for response keys, allowing us to have only one answer per trial. The details are now updated in the text.
- Figure 2B now illustrates the motion of the random dot patterns along with the corresponding response keys. The motion of the dot pattern varies around the cardinal axis used in a given block of trials (here: upward). We now clarify this in the text and in figure 2.

9) *Lines 153-154. Note that 640 successive trials in one block in the main experiment is very likely to cause fatigue in observers; 5 or 6 blocks each 120 trials, as the number of trials per block in the practice session, sounds more reasonable. More detail on the psychometric function fits would be good. For example: How many parameters? Will the upper and lower asymptote be free parameters, or fixed at 0 and 1 like in the figure? This point matters because the parameter of interest is derived from a distance between two predetermined points, it will depend in part on the asymptotes (as well as the slope).*

- Participants are allowed to take breaks within blocks at any time to avoid fatigue. We now clarified this in the manuscript.
- Further information on the psychometric function fit has been added to the text.

10) *Line 163 mentions Fig 4A depicts H1 and H0, but it does not. Figure 4B depicts hypothesis . It would make more sense to first show hypotheses about performance fields and then about attention effects; ie. 4B should be 4A and vice versa.*

- Thank you pointing that out. We have revised figure 4.

11) *Lines 165-166. These two hypotheses will be tested separately for each of the four cardinal directions. With regard to direction, I recommend exploring (a) a 2-way interaction of cardinal direction X location- RDP. For example, it is more likely that performance field asymmetries emerge when the reference axis is horizontal than vertical because overall performance would be expected to be higher at horizontal than vertical meridians and there is a slight radial/tangential asymmetry (Ezzo et al., 2023); (b) a three-way interaction among location X direction X attention condition.*

- Thank you for the suggestion. We are planning to fit a mixed effect model with subject ID as the random factor. The model will integrate both direct and interactive effects of cardinal direction, location, and attention on JND.
